# Design of a Drug-Eluting Subcutaneous Implant of the Antiretroviral Tenofovir Alafenamide Fumarate

**DOI:** 10.1007/s11095-020-2777-2

**Published:** 2020-04-15

**Authors:** Solange M Simpson, Lakmini Widanapathirana, Jonathan T. Su, Samuel Sung, David Watrous, Jiang Qiu, Elizabeth Pearson, Alex Evanoff, Dipu Karunakaran, Jorge E. Chacon, Patrick F. Kiser

**Affiliations:** 1 Department of Biomedical Engineering, Northwestern University, Evanston, Illinois USA; 2 Department of Physics and Engineering, Elon University, Elon, North Carolina USA

**Keywords:** HIV pre-exposure prophylaxis, long-acting, polyether urethanes, subcutaneous implant, tenofovir alafenamide hemifumarate

## Abstract

**Purpose:**

Sexual transmission of HIV has been clinically proven to be preventable with a once-daily oral tablet; however, missed doses dramatically increase the risk of HIV infection. Long-acting subcutaneous implants do not allow the user to miss a dose. A desirable long-acting drug-eluting implant can deliver a constant amount of drug, adjust the delivered dose, and be readily manufactured. We present a long-acting, subcutaneous implant design composed of tenofovir alafenamide hemifumarate (TAF) pellets loaded in a sealed polyether urethane tube for the prevention of HIV transmission.

**Methods:**

Implants were prepared with pressed drug pellets and extruded polyurethane tubing. *In vitro* release rate of implants using different pellet formulations, rate-controlling membranes, and geometries were measured.

**Results:**

Tenofovir alafenamide release appeared to be governed by a pseudo-steady state and followed a mass transport model of release from a cylindrical drug reservoir. Implant seal integrity was tested and confirmed using mechanical testing. The inclusion of sodium chloride in the pellet increased the release rate and reduced initial lag. The release was sustained for 100 days.

**Conclusions:**

The release rate of tenofovir alafenamide mechanistically varied with geometry and rate controlling membrane composition. The polyether urethane implant presented herein is modular and tunable to adjust the release rate and duration of the TAF release.

**Electronic supplementary material:**

The online version of this article (10.1007/s11095-020-2777-2) contains supplementary material, which is available to authorized users.

## Introduction

In 2017, 36.9 million people globally were living with HIV infection, and 1.8 million people were newly infected (1). In 2016, the Political Declaration on Ending AIDS set an ambitious goal of reducing new infections by 75% by 2020, and have achieved a 16% decrease in new infections from 2010 to 2017 (2). To accomplish this reduction of HIV transmission, the development of new technologies to stop sexual transmission will be critical. In particular, the use of antiretrovirals (ARV) for pre-exposure prophylaxis (PrEP) will be a key prevention strategy in reducing HIV acquisition in high-risk populations and quicken the pace of reducing new infections (3).

The only approved PrEP ARV is once-daily Truvada® (200 mg emtricitabine (FTC) and 300 mg tenofovir disoproxil fumarate (TDF)) (4–8). While the efficacy of Truvada® is high, efficacy decreases with decreased adherence: the VOICE trial, for example, showed approximately 4% efficacy with about 28–29% adherence (9). The need to increase user adherence has motivated the development of other routes of administration of longer-acting ARV delivery systems that provide a sustained and protective level of ARV beyond a 24-h duration. Dosing regimens ranging from once weekly to once monthly or yearly are being considered in the HIV prevention field (10).

The relationship between duration and adherence is complicated, but generally, more extended duration drug delivery systems decrease lapses in drug exposure by increasing the ease of use (11–13). The best examples of prophylactic systems that display this duration/adherence/efficacy relationship are hormonal contraceptives (14,15). Long-acting implantable contraceptives provide durable exposure to progestins up to several years and are measurably more effective than once-daily contraceptive tablets (14,16). These long-acting implants now require a visit to a physician’s office for implantation, but generally require, no follow-up until explantation (17). Similarly, long-acting ARV delivery systems offer the potential to improve adherence significantly and provide durable drug concentrations to at-risk individuals who find it challenging to take oral medication daily.

The drug formulated in a long-acting subcutaneous system must be potent and slowly eliminated, resulting in low drug doses and low volumes of distribution. To date, only a limited number of ARVs have potency and pharmacokinetic properties that permit their use in long-acting drug delivery systems with daily doses on the order of 2 mg or less. Tenofovir alafenamide hemifumarate (TAF, or GS-7340-03) is a potent prodrug of tenofovir (TFV) with a half-maximal effective concentration (EC_50_) in the low nanomolar range (5–11.2 nM) (18,19). Therefore, TAF possesses a daily dose low enough that it is reasonable to attempt to deploy it in a long-acting ARV-eluting delivery system. The phosphoramidate prodrug moiety is cleaved by caspases into intracellular tenofovir (20), which is then converted into TFV diphosphate (TFV-DP)—the active form of the drug that competitively inhibits HIV reverse transcriptase. The neutrally charged phosphoroamidate metabolically converts to negatively charged TFV-DP, which is trapped within the cell to achieve a long intracellular half-life of 150 h, thus increasing exposure (21). From an implant drug loading perspective, TAF is remarkably efficient in achieving drug exposure per unit of drug delivered. These properties make TAF one of the leading molecules for long-acting ARV delivery systems as many daily doses can be loaded inside a small controlled-release device to provide durable protection from HIV infection.

Pons-Faudoa *et al*. have recently reviewed reservoir implant designs (22). Several attempts have been made to develop subcutaneous implants that achieve the controlled release of ARV drugs for prevention. Currently, five subcutaneous implants delivering TAF appear in the literature. Gunawardana and Baum designed a drug pellet coated with poly(vinyl alcohol) and encased with a silicone scaffold (23). Perforations in the silicone scaffold expose part of the surface area of the coated pellet to the subcutaneous space. The coating and size of the exposed area control the drug release (23). Schlesinger and Desai described a heat-sealed poly(caprolactone) thin-film polymer device containing TAF and polyethylene glycol 300 at 1:2, 1:1, or 2:1 *w*/w ratios (24). The third implant, presented by Chua and Grattoni*,* consisted of a refillable titanium device that delivered TAF and FTC through silicon nanochannels (25). This refillable implant demonstrated the sustained release of TAF for 83 days in rhesus macaques (25). Johnson described a poly(caprolactone) reservoir-style device with a core formulation of TAF and castor oil and a sustained release of 0.28 ± 0.06 mg TAF/day for 180 days (26). We recently reported the pharmacokinetics and local histopathology of implants described in this paper. (27)

The published TAF implants have a core with a means of controlling the mass transport of drug from the reservoir. Other designs, e.g., Nexplanon or Mirena, have polymer occupying a significant mass fraction of the core. By having a hollow core, loading can potentially be increased, and thereby longer durations could be achieved. Ease of manufacturing and conservation of valuable drug substance are other motivations for designing hollow-core implants as the rate-controlling membrane (RCM), and the core formulation can be manufactured separately. To our knowledge, there is no publication in the peer review literature on the use of durable and elastic membranes of thermoplastics like polyether urethanes (PEU) encapsulating compressed pellets of drug substance as long-acting implants. Here, we report the design of a classical reservoir subcutaneous implant from biomedical grade polyurethanes that can deliver TAF for at least 100 days. This implant consists of a solid TAF plus excipient pellet surrounded by an RCM in the form of a tube that is heat-sealed.

## Materials and Methods

### Materials

Biomedical grade Tecoflex™ and Tecophilic™ poly(tetramethylene oxide) based poly(ether urethane) (PEU) pellets were obtained from Lubrizol Inc. (Wickliffe, Ohio), specifically Tecoflex™ EG-85A and EG-93A, and Tecophilic™ HP-60D-20. Poly(ethylene-co-vinyl acetate) (PEVA), with a 28% vinyl acetate content was obtained from Celanese Corporation (AB, Canada). TAF (GS 7340–03, CAS 1392275–56-7) and TFV or PMPA (9-(2-phosphonomethoxy- propyl)adenine, CAS 147127–20-6) were generously provided by Gilead Sciences (Foster City, CA). Sodium chloride (NaCl), magnesium stearate (MgSt), and ethanol (all USP grade) were obtained from Spectrum Chemicals (New Brunswick, NJ). Rhodamine B isothiocyanate-Dextran 70 kDa (RITC-Dx) was obtained from Sigma-Aldrich (St. Louis, MO). Ammonium acetate (LC grade), acetonitrile (LC grade), 10x phosphate-buffered saline (PBS), sodium azide (ACS grade), and methanol (LC grade) were obtained from Fisher Scientific (Fair Lawn, NJ).

### Polymer Extrusion

A detailed description of the equipment and methods used for hot-melt extrusion of PEU tubing are given in Johnson *et al*. (28). Biomedical grade Tecoflex™, Tecophilic™, and PEVA tubing of various compositions and dimensions were made by hot-melt extrusion of polymer pellets on an ATR Plasti-Corder® single screw extruder (C.W. Brabender, South Hackensack, NJ) fitted with a tubing crosshead (Guill Tool, West Warwick, RI) and appropriate tip/die combination. The polymers were first dried and assayed for water (28). PEU pellet blends were melt-mixed on a twin-screw extruder (C.W. Brabender, South Hackensack, NJ) before being pelletized and extruded to form the tubing. The polymer, outer diameter, wall thickness, zone temperatures, drawn down ratio, and draw ratio balance are outlined in the supplemental (Table S-I) for the various tubing lots used in these studies.

### Core Pellet Manufacturing

NaCl was ground using mortar and pestle and passed through a 75 μm stainless steel sieve. TAF and ground NaCl were geometrically mixed in a 98:2 wt/wt ratio, wet granulated with ethanol, and passed through a No. 40 standard testing sieve before being dried under a high vacuum (~10 Torr) with a ChemStar 1402 N pump (Welch Vacuum, Mt. Prospect, IL) at room temperature for ~24 h. 2 wt% MgSt was added to the dry formulation and roll coated. The dried granulate of 96:2:2 TAF:NaCl:MgSt was compressed into cylindrical pellets at 1000 pounds using an RD10A semi-automatic hydraulic tablet press (Natoli Engineering, St. Charles, MO). For TAF pellets used with 2.2 mm outer diameter tubing, the press was fitted with a 9.4 mm (length) × 1.8 mm (diameter) multi-compartment die and multi-tip punch (Natoli Engineering). Pellets used in implants with a 3.6 mm outer diameter were manufactured using a 10 mm (length) × 3.0 mm (diameter) die. Pellets used in implants with 0.8 cm and 1.6 cm lumen lengths were cut to 0.6 cm using a razor blade.

### Device Fabrication

**Fig. 1:**
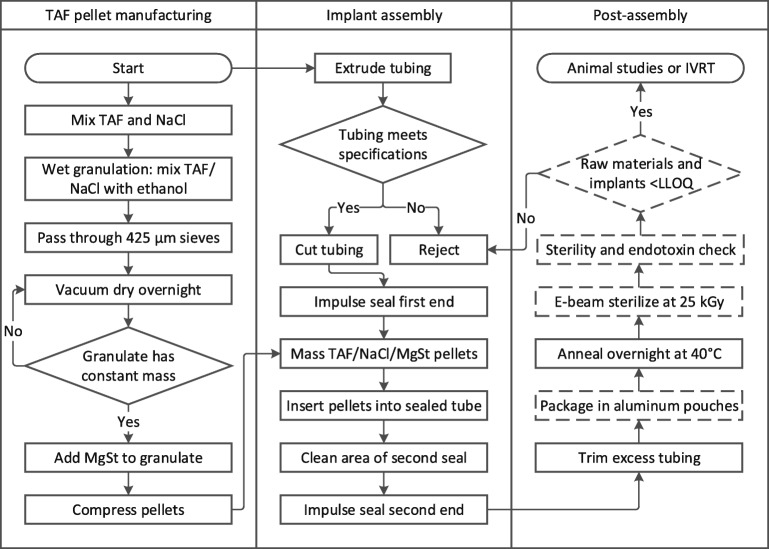
**Implant manufacturing process**. The dashed boxes in the post-assembly lane indicate processes done only for animal studies.

Figure 1 is a flowchart describing implant manufacturing processes. To assemble the implants, the PEU tubing was cut to the appropriate length, weighed, and one end was sealed using a PW2200 impulse sealer (Packworld USA, Nazareth, PA). All Tecoflex™ EG-85A TAF containing implants described herein used a sealing temperature of 120°C, sealing time of 4 s, 50% cooling from the sealing temperature, and pressure of 60 psi. The hollow tubing was then loaded with TAF containing drug pellets to the desired lumen length. The second end was sealed using the same sealing conditions and weighed. Implants were placed in metalized pouches (U-line, Pleasant Prairie, WI) and were sealed using a vacuum impulse sealer (AIE-300CA, American International Electric, City of Industry, CA). Implants were annealed for 15–20 h at 40°C to reduce any residual stress in the material after sealing (28). Finally, for sterilization studies, implants in metalized pouches underwent electron beam (e-beam) treatment with 25 kGy of irradiation (Steri-Tek, Fremont, CA). A table of all of the implants manufactured in this report is given in the supplemental (Table S-II).

### Control of Implant Variability during Manufacture

We used several controls to assure minimized implant-to-implant variability in a batch and between batches. Extrusions were conducted with an in-line laser micrometer to assure the tube outer diameter was consistent and within our targeted size constraints. Sub-batches of extruded tubing were tested by measuring the membrane thickness using a spring-loaded caliper and tubing sections within the stated dimensions ±10% were stored for further use. Pellet masses and drug strengths were measured to assure consistency of drug loading on an individual pellet basis (see supplemental section Fig. S1). Masses of pellets, tubing, and total implant masses were tracked for each implant. Finally, the lumen length of each implant was measured to assure it was within 10% of our target length.

### Characterization of Implant Seal Integrity Using High Molecular Weight Water-Soluble Polymer Conjugate

Implant seal integrity was quantified *in vitro* by the inclusion of a dry pellet containing 70 kDa water-soluble polymer conjugate RITC-Dx in the implant core. This RITC-Dx is too large to diffuse through the polymer membrane but is easily detected if the membrane wall is compromised. We set the amount of RITC-Dx loaded into the core such that the intensity of the fluorescent signal that corresponds to a 1% RITC-Dx release in one day (20 ml of *in vitro* release test (IVRT) media) of the total RITC-Dx loaded would be approximately ten times the background noise of the fluorimeter at the stated excitation and emission. Therefore, the detection of RITC-Dx in the IVRT media indicates that the implant contents are passing through defects in the seals and that the seal integrity is compromised.

**Fig. 2:**
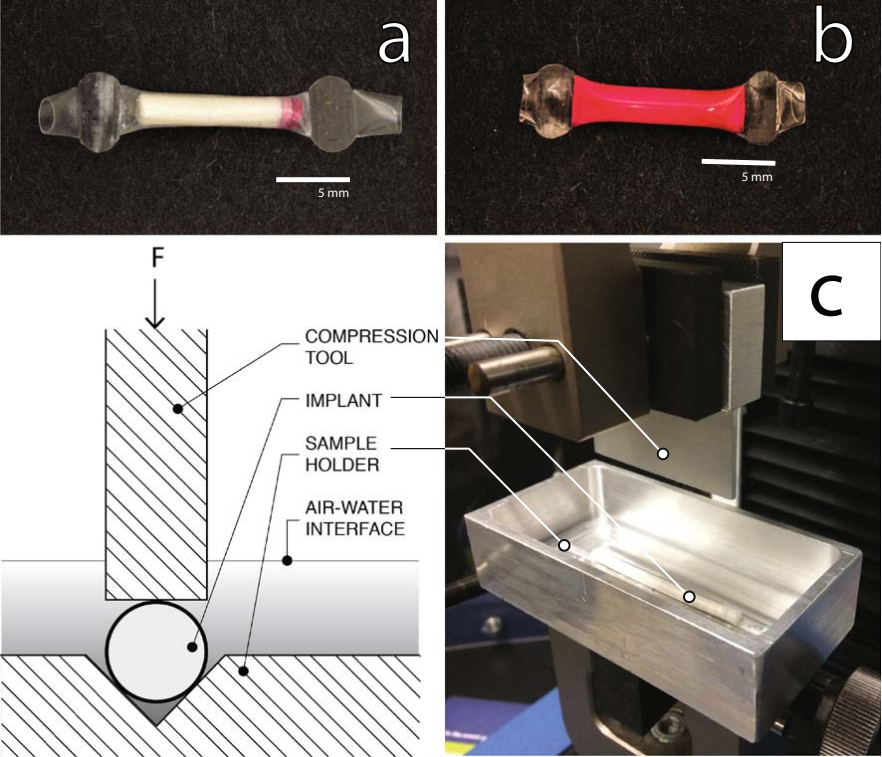
**Implant and phase 2, compression testing.** (**a**) A picture of a RITC-Dx - containing TAF implant before hydration. (**b**) A picture of RITC-Dx - containing TAF implant after hydration in PBS. (**c**) A cross-sectional view of phase 2 compression test tool and sample holder (left). Phase 2 implant mechanical testing apparatus with a compression tool and sample holder (right). The implant is placed in the V-shaped groove in the sample holder, and mechanical stress is applied by the compression tool.

RITC-Dx was compressed into 1 mm (length) × 2 mm (diameter) disks at 1000 pounds using an RD10A semi-automatic hydraulic tablet press (Natoli Engineering, St. Charles, MO). One end of the polymer tubing was sealed using the above sealing method; hollow tubing was then loaded with RITC-Dx pellets followed by TAF/NaCl/MgSt or NaCl pellets to the desired lumen length. The second end was sealed by the same method (Fig. 2a and b).

### Phase 1 Failure Testing on Test Capsules

For our initial development of sealing conditions for Tecoflex™ EG-85A, we chose three sealing temperatures (80, 110, and 140°C), three sealing times (2, 4, and 6 s), two cooling percentages (50 and 80%), and two pressures (50 and 80 psi). This design resulted in 40 parameter combinations, 32 parameter combinations with one replicate, and eight with five replicates). We made approximately 0.5 cm capsules loaded with 1 mg of RITC-Dx and 1 mg of NaCl without TAF. The test capsules were then placed in 1 ml of PBS (0.02 wt% NaN_3_). Every 3–5 days, the implants were compressed five times until hemostatic forceps handles touched (Thermo Fisher Scientific, Waltham, MA). The forceps had their jaws ground smooth and the locking mechanism removed. IVRT media was collected after each test cycle, and the percent RITC-Dx released per day was quantified by fluorescence (λ_ex_ = 525 nm, λ_em_ = 580 nm) with a SpectraMax M3 Multi-Mode Microplate Reader (Molecular Devices, San Jose, CA). From this initial screen, five seal conditions were selected for confirmation and were repeated with higher replicates (*n* = 10) using the same conditions.

### Phase 2 Compression Testing on Full TAF Implants

Because the phase 1 test does not use complete drug-loaded implants and the conditions of the phase 1 test were extreme, we needed to confirm the phase 1 conditions with prototype drug-loaded implants with a more realistic compression test. Because there would be fewer test articles to evaluate, we could use a more controlled, lower throughput computer-driven compression test. Here we wanted to use tooling to hold the implant under fluid while compressing the full length of the implant (Fig. 2c). This way, the surrounding fluid could be sampled for drug and fluorescent dye—if included— immediately after the compression test.

Implants were placed in scintillation vials with 20 ml of PBS containing 0.02 wt% sodium azide at 37°C and shaken at 80 RPM. IVRT media was changed daily and collected daily for fluorescence measurements in disposable plastic cuvettes (Fisher Scientific, Fair Lawn, NJ). Percent RITC-Dx released per day was quantified by fluorescence (λ_ex_ = 525 nm), λ_em_ = 580 nm) and was measured with a Shimadzu RF-6000 spectrofluorometer (Shimadzu Corp., Kyoto, Japan).

The phase 2 compression test was performed in a V-shaped sample holder (6063 aluminum) with varying sizes of compression tools (6061 aluminum) (see Fig. 2c). The V-shaped well was used to hold implants in place during testing. Given that implant lumen lengths ranged from 1 cm to 5 cm, tools of different sizes (0.5 cm to 5 cm in length and 2 mm to 3 mm in width) were made to allow flexibility in our mechanical testing protocols. Immediately before the application of mechanical stress, IVRT media was collected to measure baseline TAF release and fluorescence. Implants were then placed in 10 ml of fresh IVRT media and subjected to a cyclic compressive load using an Instron 3342 force transducer (Instron, Norwood, MA) and the compression tool. First, the load cell approached the implant at 2 mm/min until the load cell applied a 0.03 N force, which indicated to the force transducer that the compression tool was touching the implant. This state triggered the start of the compression test. Then implants were compressed ten times by a distance of 1.1 mm (50% compression) at 40 mm/min (Fig. 2c). After the test was complete, media samples were collected to measure drug and fluorescence. Implants were returned to 20 ml scintillation vials, and IVRT media samples were collected to measure fluorescence and TAF release for two subsequent days in case failure was missed during the time of the mechanical stress test. The above test was performed three times between 13 and 15 days, 20–22 days, and 27–30 days from the beginning of IVRT. We applied stringent criteria for confirming seal integrity by setting a threshold of less than 1% of the cumulative RITC-Dx load after three phase 2 mechanical stress tests and 91 days on IVRT.

### HPLC Analysis of TAF

The amount of TAF released at each time point was determined with an Agilent 1200 series HPLC (Agilent, Santa Clara, CA) equipped with a diode array detector. A Zorbax Eclipse Plus C18 column (4.6 × 100 mm, 3.5 μm, Agilent) and a Zorbax Eclipse Plus C18 guard column (4.6 × 12.5 mm, 5 μm, Agilent) were used. For TAF detection, a mobile phase A (20 mM ammonium acetate) and mobile phase B (acetonitrile) were pumped (gradient t = 0: %A = 100, %B = 0, t = 8: %A = 45, %B = 55, t = 9.6: %A = 45, %B = 55, t = 9.7: %A = 100, %B = 0, t = 12: %A = 100, %B = 0) at a flow rate of 0.7 ml/min with the column oven temperature set to 25°C. A 50 μl aliquot of sample was injected, and the column effluent was detected at 260 nm with a UV detector. The total elution time was 12 min.

Because TAF converts to other species in solution (29), the hydrolysis kinetics of 1 mM TAF in PBS was measured in triplicate over 80 h at 37°C. Roughly 24% of TAF converted to PMPA monoamidate after 22 h in pH 7.4 PBS. TAF hydrolysis displays pseudo-first-order hydrolysis kinetics near pH 7.4 (See supplemental fig. S2). We estimated from the first-order rate constant (2.85 X10^−6^ s^−1^) a solution half-life of TAF of 2.8 days in our IVRT media. Therefore, TFV-related species, PMPA monoamidate and monophenyl PMPA were also quantified with TAF parent and TFV. The sample concentration was determined using calibration curves prepared with TAF (40–800 μg/ml) standards used to quantify TAF parent (GS-7340) and monophenyl PMPA and TFV (10–100 μg/ml) standards to quantify TFV, and PMPA monoamidate injected at 1–10 μl volumes.

### *In Vitro* Release Testing

The solubility of TAF was measured by dissolving an excess quantity of TAF in PBS at pH 7.4 and 37°C with shaking at 80 RPM. The kinetics of dissolution was measured by filtering the solution and measuring the TAF concentration by HPLC. The concentration reached a plateau after 12 h. The maximum solubility of TAF was measured to be 10.5 mg/ml in PBS. A typical concentration of TAF detected in PBS IVRT media was on the order of 10 μg/ml; therefore, all in vitro release studies were conducted under sink conditions. For implant sets with 3.6 mm outer diameter and 3.7 cm lumen length, implants were placed in 25 ml of buffer due to the increased length. 0.02% *w*/*v* sodium azide was added to PBS to prevent bacterial growth that may otherwise be supported by PBS over a long IVRT experiment. TAF in vitro release from the implants was measured in 20 ml of IVRT media (PBS with 0.02% w/v sodium azide) at 37°C and shaken at 80 RPM in an I26 Incubator/Shaker (New Brunswick Scientific, Edison, NJ). IVRT media was changed daily to maintain sink conditions. Samples were collected for HPLC analysis on days 1, 2, 3, 5, 7, 10, 14, and every seven days after. Samples were stored at −80°C until the HPLC runs were conducted.

### Extraction of TAF from Implants

Implants that had completed 30, 45, or 60 days of IVRT were removed from release media, carefully dried, transferred to a clean scintillation vial, and frozen at −80°C. To minimize drug loss, frozen samples were individually weighed and ​laterally cut inside an aluminum weighing dish. Contents from the weigh dish were quantitatively transferred into ​a 25 ml volumetric flask ​and diluted to volume using methanol. The ​resulting solution was diluted 1:10 (*v*/v) into a 10 ml volumetric flask using methanol ​and filtered through a 0.2 μm nylon syringe filter into an HPLC vial for analysis using the ​described HPLC method for IVRT.

### Measures of TAF Release and Mass Balance

Because TAF hydrolyzes at pH 7.4, we tracked the concentration of several TAF related species (29). For measures of drug release, we express drug release as the mass of TAF accumulated in the IVRT media every 24 h period. The actual amounts released are higher than what we report as the released tenofovir alafenamide hydrolyzes in the external IVRT media while the sample is waiting to be collected (see fig. S1). Often we use average release rates as a measure for comparison of formulations. Because implants of this type have a start-up period where the release rate is changing rapidly, we wanted a release measure that captured the plateau release rate value. We found numerically the rate of change of the release rate typically leveled off near day 7 and in some implants release rate again changed rapidly on day 91 as most implants became exhausted of drug. Therefore we measured average drug release rates over days 7–91 as a comprehensive measure of implant delivery of TAF.

For similarity analysis and other measures of cumulative drug release that require mass balance we use a measure of TAF equivalents *m*_*TAF*, *t*_shown in eq. 1, in which all of the species present in the IVRT media are quantified and used to compute the mass of TAF that has been depleted by release into the external media:

1 $$ {m}_{TA F,t}=\left\lceil {n}_{TA,t}+{n}_{PMPA1,t}+{n}_{PMPA2,t}+{n}_{TFV,t}\right\rceil\ {M}_{TA F} $$

Where n_TA,t_ is moles of tenofovir alafenamide at time t, M_TAF_ is the molecular weight of TAF, and the indexes are PMPA1: PMPA monoamidate, PMPA2: monophenyl PMPA. The time course of this value can be integrated to determine the fraction of molecules as a function of time that has been released from the core of the device. Finally, eq. 2 shows that for cylindrical implants of similar geometry, mass transport will be proportional to the length *L*. Therefore by normalizing the release rate to unit length, formulations that are of variable length can be directly compared as TAF unit length release rate (μg/cm/day).

### Molecular Weight Analysis of PEU

Polymer tube samples were analyzed before and after e-beam sterilization. The PEU tube material was dissolved in tetrahydrofuran and filtered through a 0.2 μm PTFE syringe filter before analysis. Gel permeation chromatography (GPC) (Wyatt Technology, Santa Barbara, CA) was then used to measure the molecular weight of the tubing. The GPC system consisted of an Agilent 1200 HPLC (Agilent, Santa Clara, CA) with refractive index (Optilab T-rEX, Wyatt Technology, Santa Barbara, CA) and a multi-angle light scattering detector (DAWN HELEOS-II 8 angle, Wyatt Technology, Santa Barbara, CA), and the data were processed with ASTRA software (Version 7.0). The separation columns used were a PLgel guard column (10 μm, 7.5 × 50 mm) and GPC column PLgel Mixed-B column (10 μm, 7.5 × 300 mm) at 25°C. The flow rate of the mobile phase, tetrahydrofuran, was 0.7 ml/min, and the injection volume was 100 μl.

### A Steady-State Model of Implant Drug Release

Steady-state drug release rates from ideal cylindrical reservoir devices can be described using a simple transport model (30–32):

2 $$ \frac{d{M}_t}{dt}=\frac{2\pi LDK\varDelta C}{\ln \left(\frac{r_o}{r_i}\right)} $$

Where *dM*_t_/*dt* is release rate, *L* is length along which diffusion occurs, *D* is the diffusion coefficient, *K* is the partition coefficient between the membrane and core, *△C* is the concentration difference between the inner and outer radius, *r*_o_ is the outer radius, and *r*_*i*_ is the inner radius. This model assumes a constant diffusion coefficient, constant volume, zero drug concentration at the outer wall, and steady-state conditions.

### Similarity Factor Calculations

The similarity (f2) factor was calculated (Eq. 3) to compare the effects of e-beam sterilization and thermal, long-term storage on these implants. The value was calculated using the following equation according to FDA guidance on comparing dissolution data (33,34):

3 $$ {f}_2=50\ast \log \left(\frac{100}{\sqrt{1+\frac{\sum_{t=1}^n{\left({R}_t-{T}_t\right)}^2}{n}}}\right) $$

Where R_t_ is the cumulative release at time t of the reference batch, T_t_ is the cumulative release of the test batch at time t, and n is the number of overlapping time points. The cumulative release was calculated using the trapezoidal rule to estimate the area under the curve. According to the FDA guidance, f2 above 50 ensure equivalence of the *in vitro* performance of the unstressed test and reference batches (33).

### Implant Dimension Constraints

Implant dimensions were guided by dimensions of implants that are approved products. Norplant™ contraceptive implants consisted of six 2.4 mm diameter, 3.4 cm long rods (35). Norplant-II™ contraceptive implants consisted of two 2.4 mm diameter 4.4 cm long rods (35). Implanon™ is a single rod 2 mm in diameter and 4 cm long (22). The Supprelin™ LA and Vantas™ histrelin acetate implants are 3 mm diameter and 3.5 cm long (22). Viadur™ subcutaneous implants are 4 mm in diameter and 4.5 cm long, with larger implants being claimed as practical (36). Thus, the design space for subcutaneous implants ranges from 2 to 4 mm in diameter and 4–4.5 cm in length.

## Results

### Sealing conditions and mechanical stress testing

At least four experimental factors define validatable thermal impulse polymer-welding: temperature of the heating element, the time that the heating element is held against the substrate, the pressure that the jaws apply to the two leaflets of the substrate, and the time the substrate is allowed to cool before it is released by the impulse sealing instrument. We found that each polymer membrane formulation requires an optimization step to assure high integrity seals are produced when designing this type of implant. To consistently explore the impact of these four experimental factors on seal integrity, we needed to construct somewhat large experimental designs, especially when several replicates are present. Therefore, we took a two-phase approach to identify implant sealing conditions that resist rupture and dose dumping. In phase 1, we would explore multiple sealing conditions on many ‘test capsules’ and attempt to force failure in the test article. In phase 2, we would confirm that the conditions identified in phase 1 generated robust seals via a compression test on complete TAF loaded implant prototypes using an electromechanical testing instrument.

For the test article, we considered using drug-loaded implants in phase 1 tests but decided against this to conserve drug substance. We chose to label the internal compartment of the phase 1 test capsule with a non-releasable fluorescent molecule (RITC-Dx) that can be readily detected when leaks occur. We included an osmotically active agent (NaCl) in the test capsule that would cause the article to imbibe fluid and pressurize. Swelling applies background stress to the test capsule (much like the full implant). We sought to make the phase 1 mechanical failure test significantly more extreme than what would be experienced by an implant *in vivo* or during typical *in vitro* testing. We hypothesized that if a particular sealing condition could produce a capsule that could survive multiple extreme phase 1 failure tests, the corresponding drug-loaded implant would likely remain intact *in vivo* and during *in vitro* tests.

In the development of the phase 1 test, we found that we could reproducibly compress an impulse sealed PEU capsule with a pair of 7 in. modified hemostatic forceps. The test capsule was seized within the jaws of the modified hemostat parallel to the length of the capsule. The capsule was compressed five times in quick succession with maximum hand-applied force. We ground ridges on the jaws off the clamping surface to prevent damage to the polymer membrane, and we removed the locking mechanism of the hemostat to allow repeated and rapid compression. This compression would cause a short 0.5 cm test capsule to undergo a large deformation of the polymer at the interface between the internal contents and both seals. A short test article tended to focus the deformation at the seal rather than along the length of the capsule, making the test even more extreme.

For each polymer used, we first had to determine the minimum and maximum temperatures and times used in the phase 1 sealing condition scan. We conducted a scan of sealing conditions with varying combinations of sealing temperature (T_s_, °C), sealing time (t, s), % cooling of sealing temperature (%T_s_, %), and pressure (P, psi). In this full scan, we formed test seals on blank polymer tubes for increasing times and temperatures. This allowed us to define a minimum temperature and time for melting, and maximum temperature and time where the polymer was overheated: not thinned below ~100 μm, discolored and showed the absence of bubbles formed in the polymer melt that is indicative of degradation. With the minimum and maximum temperatures, a four-factor experiment design was constructed varying times of heating, time of cooling, temperature, and jaw pressure.

**Fig. 3:**
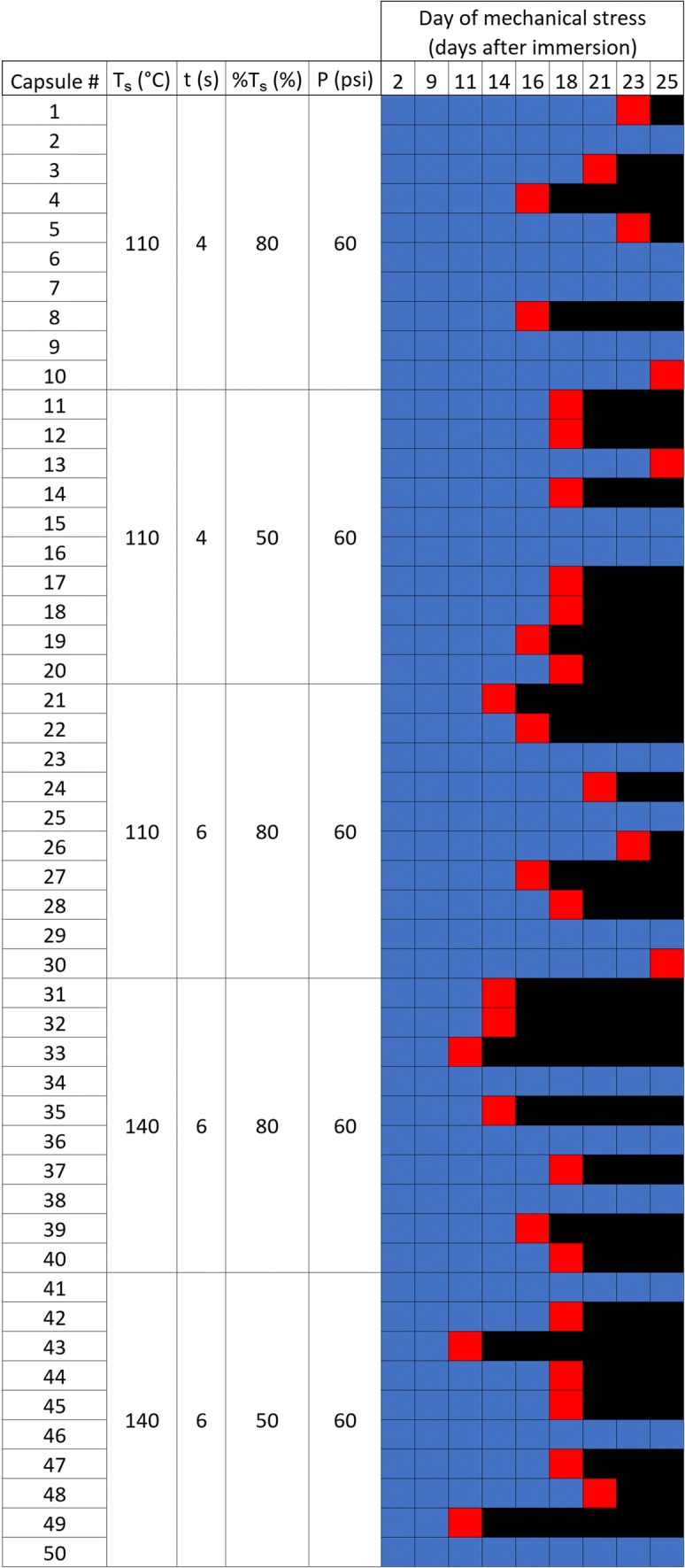
**Representative heat map used to find working sealing conditions using the phase 1 failure test.** 10 test capsules for each sealing condition is displayed in the heat map. Each colored square represents an individual implant test**.** Blue boxes represent implants that have intact seals after 5 complete compression cycles; red boxes represent implants leaking >10% of RITC-Dx, and black boxes represent no further data was collected because the capsule failed.

Figure 3 shows five sets of sealing conditions across ten identical test capsules. We found the condition T_s_ = 110°C, t = 4 s and %T_s_ = 50% at *P* = 60 psi generated implants that passed our phase 1 failure test. These sealing conditions were then used to manufacture larger implants with TAF included in the core. We note, other sealing conditions in this experiment also gave implant sets that met our phase 1 test selection standard. We subsequently manufactured hundreds of implants composed of the PEU Tecoflex™ EG-85A for subsequent studies using these sealing conditions, with no detected evidence of dose dumping, leaking, or implant failure *in vitro* or *in vivo* (27) or during any phase 2 mechanical stress test applied to them.

### Formulation development

**Fig. 4:**
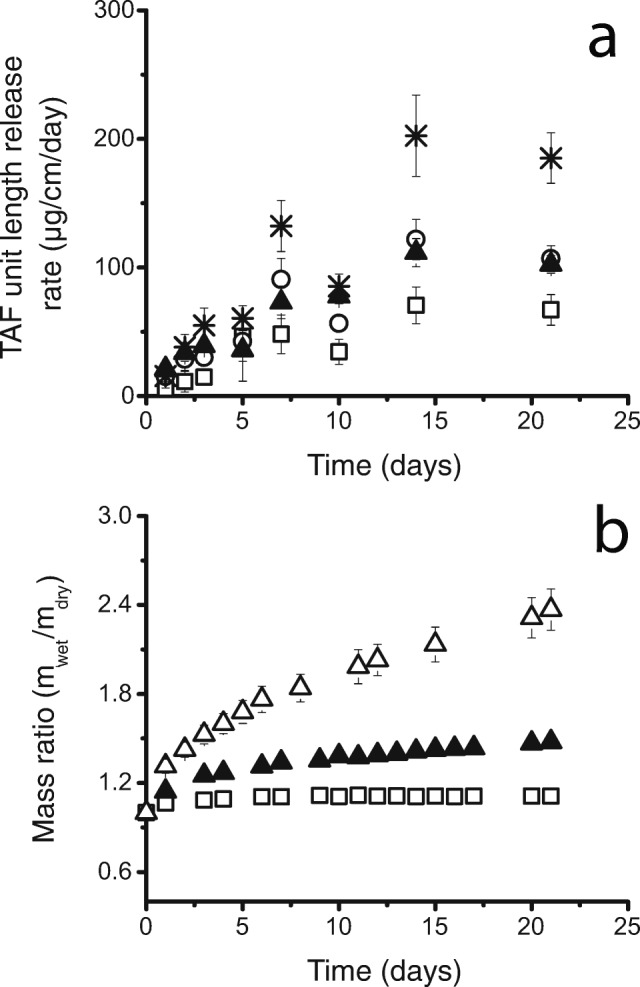
**The effect of increased NaCl concentration. **(**a**) Average daily release of TAF per unit length from implants made with a core formulation of TAF wet granulated with 0% (□), 1% (○), 2% (▲), and 5% (*) NaCl. Implant RCM is made from Tecoflex™ EG-85A (2.2 mm OD, 150 μm thickness, 10 mm lumen length; 8 mm lumen length for 2% NaCl). Error bars represent standard deviation (*n* = 10). (**b**) The average mass swelling ratio of implants with core formulation of TAF wet granulated with 0% (□) and 2% (▲) NaCl and NaCl alone with the same mass in the 2% formulation (∆). 2 wt% MgSt was added to each dried formulation. Error bars represent standard deviation (*n* = 5).

The PEU that forms the RCM in these implants has hydrophobic soft-blocks composed of water-insoluble poly(tetramethylene oxide) subunits (37) that cause these PEUs to be lipophilic. However, these polymers swell slightly (~3% by weight) in aqueous media, and this work shows that water can conduct through the hydrophobic PEU membrane. Figure 2a shows a typical test implant with a TAF pellet, a RITC-Dx pellet, and impulse sealed ends. This ability to mix with water, coupled with the short transport distance of 200 μm or less, allows water to enter the core of the implant and hydrate the core contents (see Fig. 2b). This influx of water wets the drug pellet in the core of the device and generates a suspension of solid drug substance surrounded by a TAF solution that is likely saturated or near-saturated in the core of the device. This concentration gradient drives drug release. Over about a week, the implant nears swelling equilibrium (Fig. 4b).

We found it advantageous to include an osmotic attractant like NaCl to accelerate the hydration of the TAF pellet, thereby reducing the duration of the lag period in the release curve. Figure 4a displays the average TAF release *versus* time for up to 5 wt% NaCl addition. We observed an increase in swelling and implant bursting with the addition of 10 wt% NaCl in the pellet. Therefore, we limited the amount of NaCl to 5 wt% in the pellet. There was little difference in release rate between 1 wt% and 2 wt% NaCl loading. The average TAF release rate was highest in implants loaded with 5 wt% NaCl at 187 ± 57 μg/cm/day and was lowest in the implants with no NaCl included. By day 63, the implants with an addition of 0, 1, 2, and 5 wt% NaCl released a total of 17 ± 2%, 26 ± 1%, 32 ± 1%, and 56 ± 6% (*n* = 10, ±SD) TAF of the initial loading. We observed the implants with 5% NaCl loading to be more swollen than the implants with 1 wt% and 2 wt% NaCl loading. The increase in swelling with increased NaCl loading is quantified by the ratio of swollen mass to dry mass over time, comparing implants with 0 and 2 wt% NaCl loading (Fig. 4b).

**Fig. 5:**
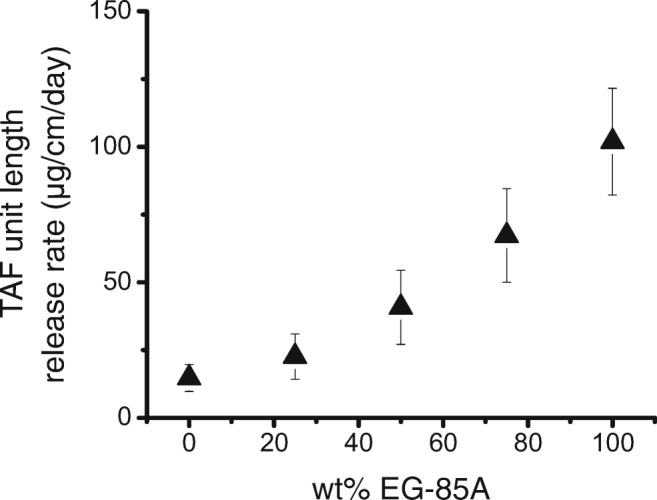
**The effect of PEU blend composition on the release rate.** Average daily release of TAF equivalent per unit length from implants made with varying RCM polymer composition (2.2 mm OD, 150 μm thickness, 10 mm lumen length; 8 mm lumen length for 100% EG85A). Tecoflex™ EG-93A makes up the remaining percentage of the RCM polymer composition. Core formulation is 96:2:2 TAF:NaCl:MgSt. RITC-Dx is incorporated in the core to measure the seal robustness. Error bars represent standard deviation (n = 10 for all except 25% EG-85A (*n* = 7) and 0% EG-85A (*n* = 3)). The average was over days 7 to 91 for each set.

We constructed TAF implants with similar core compositions and comparable geometries, but variable RCM PEU compositions. Figure 5 demonstrates a positive nearly linear relationship between release rates and increasing percentages of Tecoflex™ EG-85A to Tecoflex™ EG-93A (Pearson correlation coefficient, r = 0.973). Table I shows the average release rates of TAF as a function of the material composition of the implant membrane. A single hydrophilic PEU was chosen for the screen with increased hydrophilic properties possessing PEG and poly(tetramethylene oxide) (PTMO) soft blocks forming a hydrophilic PEU membrane (Tecophilic™ brand PEU) (Table I). Membranes made of 12.5: 87.5 (*w*/w) Tecophilic™ HP-60D-20: Tecoflex™ EG-85A had an average TAF release rate of 95 ± 23 μg/cm/day. The hydrophilic PEU did not provide a release rate above the EG-85A membranes. PEVA, which has a long history of use in contraceptive drug delivery, was also evaluated. However, TAF release through PEVA was negligible.

**Table I:** **The Average Release of Implants with Increasing Polymer Hardness.** The Average Release was Determined by Calculating an Average of TAF Days 7 to 91, and the Error was Determined by Averaging the Standard Deviation of All Points from Days 7 to 91. All Membrane Thicknesses Were 150 μm

Polymer membrane composition	TAF unit length release rate^a^ (μg/cm/day)
Tecoflex™ EG-85A: Tecophilic™ HP-60D-20 87.5:12.5	95 ± 23
Tecoflex™ EG-85A	102 ± 20
Tecoflex™ EG-85A: EG93A 75:25	67 ± 17
Tecoflex™ EG-85A: EG93A 50:50	41 ± 14
Tecoflex™ EG-85A: EG93A 25:75	23 ± 8
Tecoflex™ EG93A	15 ± 5
PEVA 28% vinyl acetate	0.6 ± 1

^a^Average release rate of TAF on days 7–91

### Use of a steady-state drug release model in implant design

**Fig. 6:**
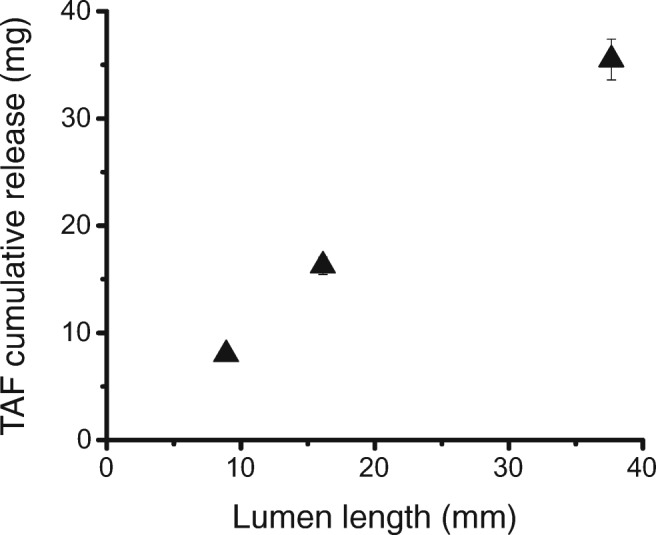
Cumulative release *vs*. lumen length. Implants composed of Tecoflex™ EG-85A RCM (2.2 mm OD, 150 μm thickness) with 0.8, 1.6, and 3.7 cm lumen lengths. Core formulation is 96:2:2 wt% TAF:NaCl:MgSt. Error bars (± SD) (*n* = 10 for 0.8 cm and 3.7 cm; *n* = 9 for 1.6 cm).

Ideal reservoir implants, once steady-state is reached, should follow eq. 2 as a mass transport model derived from Fick’s Law. Therefore, experiments were designed to determine if the drug release scaled according to this steady-state model. We first compared release rates normalized to the lumen length across three different lumen lengths: 0.8 cm, 1.6 cm, and 3.7 cm. Figure 6 shows the cumulative release *vs*. the lumen length, demonstrating that the release rate scales linearly with lumen length. All implants were studied for at least 91 days, so this experimental duration was used as a basis of comparison. The TAF unit length release rate to the lumen length for each set of implants was 102 ± 20, 112 ± 24, and 105 ± 26 μg/cm/day, respectively, averaged over days 7 to 91. A two-sample t-test assuming equal variances was performed for implants with 0.8 cm lumen length and 3.7 cm lumen lengths, and it was found that the difference in mean release rates was not statistically significant (*p* > 0.05). In total, these data imply that an end effect has a negligible contribution to the release rate of the implants studied.

**Fig. 7:**
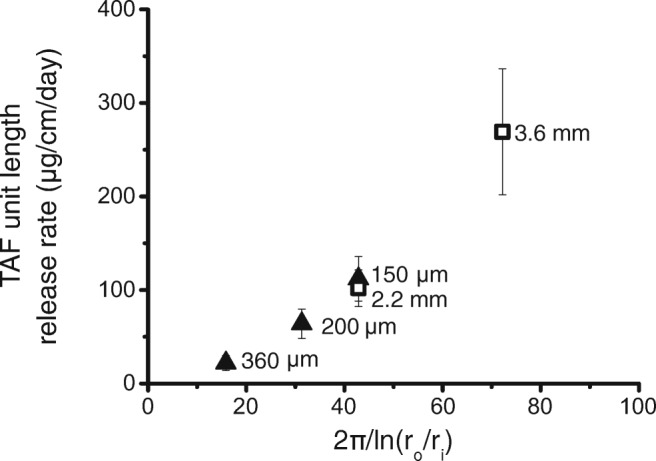
**Average TAF unit length release rate*versus*2π/ln(r**_**0**_**/r**_**i**_**) for implants made with varying RCM thickness (**▲**) and varying outer diameter (**□**).** All implants were made using Tecoflex™ EG-85A with a core formulation of 96:2:2 TAF:NaCl:MgSt. There were three groups of implants with 2.2 mm outer diameter implants with varying RCM thicknesses of 150, 200 and 360 μm RCM thickness (▲), and two groups of implants with a 2.2 mm and 3.6 mm outer diameter implants with 150 μm RCM thickness (□). The average *in vitro* release rates per unit length is an average over days 7 to 91. Error bars represent standard deviation (n = 10; *n* = 9 for 2.2 mm outer diameter).

The model also predicts that mass transport will be proportional to the inverse of the natural logarithm of the ratio of the outside to the inside radii of the implant. Figure 7 shows the change in the release rate per lumen length based on the change in the relationship between the outer and inner radii of the implant. There were three membrane thicknesses compared: 150 μm, 200 μm, and 360 μm all with a 2.2 mm outer diameter, and there were two outer diameters compared: 2.2 mm and 3.6 mm both with a 150 μm wall thickness. There is a positive linear relationship between the release rate and 2π/ln(r_0_/r_i_), suggesting that the model can be used to estimate the pseudo-steady-state release rate of a theoretical TAF implant with new dimensions after one measures the TAF release rate of a single polymer composition and core formulation (Pearson correlation coefficient, r = 0.982).

### Physical stability of the PEU TAF implants

Because implants were sterilized for animal PK and safety studies, we verified that e-beam sterilization did not greatly influence the performance of the implants. Radiation-induced changes in the membrane and drug recovery were assayed by measuring the implant release rate and TAF strength before and after sterilization. The average TAF mass recovered from extracted implants was 58.05 ± 2.19 mg for the unsterilized implants, and 59.02 ± 1.32 mg for the e-beam sterilized implants. Release curve similarity f2 was 53.3, indicating that the unsterilized and e-beam sterilized implants demonstrated equivalent release profiles (see supplemental fig. S3). The average TAF release rate was 93 ± 17 μg/cm/day (*n* = 5, ±SD) for unsterilized implants and 112 ± 24 μg/cm/day (*n* = 9, ±SD) for sterilized implants. By day 91, the unsterilized and sterilized implants had a significant difference in average TAF cumulative release of 39 ± 2% and 48 ± 3%, respectively (*p* < 0.05). Unsterilized TAF implants (n = 5) had an average TAF % recovery of 95.24 ± 0.01% and TFV % of 0.50 ± 0.00% of the total pellet weight, and the sterilized TAF implants (n = 5) had an average TAF % recovery of 95.09 ± 0.01% and TFV % recovery of 0.50 ± 0.00% of the total pellet weight.

The weighted average molecular weights (M_w_) and the number average molecular weights (M_n_) of the Tecoflex™ EG-85A were 92.64 ± 2.03 kDa and 46.33 ± 1.34 kDa, respectively for unsterilized implants from the same batch, and the average M_w_ and M_n_ were 96.01 ± 2.08 kDa and 44.71 ± 1.41 kDa, respectively for the e-beam sterilized implants. The average polydispersity index values (M_w_/M_n_) were 2.001 ± 0.087 for the unsterilized implants, and 2.149 ± 0.028 for the e-beam sterilized implants.

The reproducibility of the release curve was also assessed under heat stress as previous polyurethane intravaginal rings showed significant changes in drug flux after thermal stress (32). Implants were stored in heat-sealed metalized pouches at 40°C for six months, room temperature (20–25°C) for one year, and − 20°C for one year. The implants stored at these conditions were compared to implants immediately placed on IVRT. The similarity factor f2 for implants stored at 40°C for six months was 77.3, and the f2 for implants stored at room temperature for a year was 71.2. Therefore, the stressed implants demonstrated equivalent release profiles to implants that were immediately placed on IVRT (see supplemental fig. S4).

### Implants selected for PK studies and TAF related substances

**Fig. 8:**
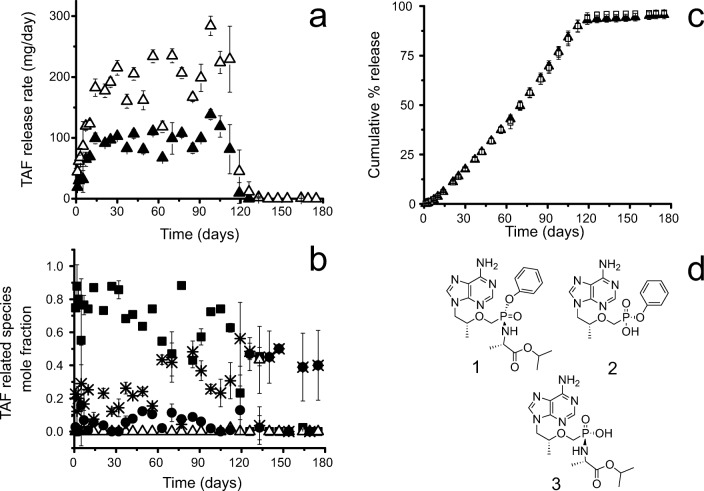
(**a**) Average daily release of TAF equivalent from 0.8 cm, 16.8 mg TAF (▲), and 1.6 cm 33.9 mg TAF (∆) implants used in animal PK and safety studies. Implants were made with Tecoflex™ EG-85A RCM (2.2 mm OD, 150 μm thickness) with a core pellet composition of 96:2:2 wt% TAF:NaCl:MgSt. Error bars represent standard deviation (n = 10, n = 9 for 1.6 cm implant). (**b**) Average daily release of TAF parent (■) and TAF related species; PMPA monoamidate (*), monophenyl PMPA (∆) and TFV (●) from the same 0.8 cm implant above. Error bars represent standard deviation (n = 10). Numeric values given in Table S-III. (**c**) Cumulative % release of TAF equivalent from 0.8 cm (▲) and 1.6 cm (□) implants. Error bars represent standard deviation (n = 10, n = 9 for 1.6 cm implant). (**d**) TAF parent (1) is tenofovir alafenamide without the fumarate salt, which undergoes pH-dependent hydrolysis into two main related substances monophenyl PMPA (2) and PMPA monoamidate (3). These are the Generation A implants of the recent Su *et al*. (27).

With these data in hand, we designed a TAF implant whose pharmacokinetics could be evaluated in an animal model (27). Figure 8 shows the release rates of representative implants and the mole fraction of TAF related species *versus* time of 0.8 cm lumen length implants. By the time the implants were no longer releasing drug, the implants with 0.8 cm and 1.6 cm lumen lengths had an average TAF equivalent cumulative release of 95 ± 2% and 96 ± 1%, respectively, of the initial TAF loading. One complication of working with TAF in long-acting dosage forms is the hydrolysis of the parent compound. Over the first 24 h of collection early in the release curve, we observed the prodrug to hydrolyze into about a 9:1 molar ratio of TAF to a related substance in the PBS IVRT media (Fig. 8b). Over the long IVRT time the mole ratio of PMPA monoamidate increased in the IVRT media such that there was an average of 20 ± 6 mol% PMPA monoamidate over days 1 to 56 with a noticeable increase at day 63 at an average of 35 ± 15 mol% from days 63 to 126 (Fig. 8b). See Table S-III for the daily amount of all species detected in the IVRT media for an implant of 0.8 cm Tecoflex™ EG-85A RCM (2.2 mm OD, 150 μm thickness) with a core pellet composition of 96:2:2 TAF:NaCl:MgSt and a TAF loading of 16.8 mg. The saturation concentration of TAF was measured to be 10.5 mg/ml in PBS at 37°C. We examined the contents of the implant core over time (Table II) while on *in vitro* release. Over 60 days, approximately 88% of the TAF loaded is present in the core, mostly as solid drug. Therefore, the interior of the implant rapidly becomes saturated, and the vast majority of the drug inside the implant exists in solid form.

**Table II:** Analysis of Internal Contents During *In Vitro* Release Testing

Time (day)	% TAF^a^ released (wt%; means ± SD)^b^	Wt% TAF remaining in the implant^f^ (wt%; means ± SD)^c^	Wt% monophenyl PMPA in implant (wt%; means ± SD)^c^	Extracted mass of TAF in the implant (mg; means ± SD)^c^	Purity of TAF in the implant (mol %; mean ± SD) ^c^
0	0.0 ± 0.0	100	0	16.2 ± 0.4	100
30	17.5 ± 0.8	70.4 ± 4.7	4.8 ± 0.4	11.4 ± 0.7	91.5 ± 0.2
45	29.1 ± 1.4 ^d^	57.7 ± 2.8	4.9 ± 0.1	9.4 ± 0.6	89.6 ± 0.5
60	40.2 ± 2.0 ^e^	50.2 ± 2.6	5.1 ± 0.4	8.2 ± 0.5	88.0 ± 0.9

^a^TAF equivalent

^b^*n* = 10

^c^n = 3

^d^Average of days 42 and 49

^e^Average of days 56 and 63

^f^150 μm EG-85A with a core formulation weight ratio of 96:2:2 TAF:NaCl:MgSt; swollen implant core volume was ~350 μL

## Discussion

In our first attempts at making PEU based TAF implants, we found that the implants had osmotically swelled, causing implants made by multiple sealing techniques to fail. We also observed a tendency for transient dose dumping *in vitro* when a seal failed. *In vivo* seal failure could lead to unpredictable dose dumping, high local drug exposure, and possibly local toxicity *in vivo*. For this reason, finding robust sealing conditions was imperative to the development of this type of implant. To mitigate this risk, we developed stress testing methods to aid in searching for robust sealing conditions. We found that our coupled phase 1 and phase 2 failure testing methods (Figs. 2 and 3) were an effective empirical way to find reliable sealing conditions and study seal performance over time. Using this testing scheme, we did not observe a single implant failure over hundreds of implants under IVRT or in animals (data not shown). We also found that sealing conditions are not necessarily transferable from one thermoplastic formulation to another. The difference in melt behavior of each polymer formulation required us to scan sealing conditions to develop robust implants that do not leak during testing. It is not apparent from published reports if other TAF reservoir implant designs also suffer from seal integrity issues.

We began this work speculating that we would need to use a hydrophilic PEU to deliver the water-soluble salt TAF (e.g., Lubrizol Tecophilic™ or AdvanSource Hydrothane™ brands). As we have reported previously, hydrophilic PEUs are capable of delivering TFV (28) and TDF (38) from a thicker-walled vaginal ring. Prototype implants made of these hydrophilic PEUs released TAF too rapidly. We found that a much more hydrophobic PTMO based PEU could create functioning implants that allowed water to penetrate the membrane, dissolve TAF, and then allow conduction of the drug molecule across the membrane.

We chose to investigate this modular pellets-in-a-tube design because it provided ample flexibility in evaluating implant design inputs. Furthermore, as drug loading is critical to achieving long durations, a compressed pellet could have high drug loading per unit volume of the implant using this design. We achieved drug loading of approximately 740 mg of TAF per ml of core volume or 16.8 mg in 0.023 ml tube internal volume. We avoided hot melt coaxial extrusion because of drug stability issues of the TAF prodrug at the temperatures required for PEU extrusion.

The pellets-in-a-tube design has several drawbacks that provide a basis for design improvements. The design is simple in principle, yet in comparison to the continuous coaxial extrusion manufacturing like that used in PEVA devices, the TAF implants have a more complex manufacturing process (see Fig. 1). Furthermore, the pellets are made by a hand press and not by a high-speed pellet press. The drug loading density of the devices is high, but in practice, one cannot easily match the drug densities generated with coaxial extrusion where the thermoplastic process can exclude air and achieve drug densities higher than a compressed pellet made on a standard pellet press. The pellets are hard enough to be able to pick them up with forceps and insert into the PEU tubing, but also are more friable than typical tablets that have a higher mass-fraction of tablet excipients. Implying further engineering will need to be performed on the pellets to manufacture large batches of this type of system.

Our studies show that these PEU-based TAF implants are tunable reservoir implants where flux and the daily dose of TAF can be readily adjusted (Fig. 7). We demonstrate that TAF release rates scale linearly with membrane surface area, which aligns with Schlesinger *et al*. They found that this is true when TAF is formulated with PEG300 but not for TAF alone (24). We similarly confirm that the TAF release rates scale linearly with membrane surface area in the 2% NaCl formulations. A long lag-time is undesirable because users would have to wait longer to reach protective ARV concentrations. We found we could shorten the lag-time for the implant to approach a pseudo-steady-state release rate by adding NaCl to the formulation. The addition of NaCl to the formulation not only reduced the lag-time but also increased the release rate of the implants. We constructed TAF with variable RCM PEU compositions. Increasing elastic modulus is associated with increasing crystallinity of the PEU and reduced drug diffusivity (39). Tecoflex™ EG-85A and EG-93A have elastic moduli at 100% strain of 4.1 MPa and 6.9 MPa (40), reflecting an increased volume fraction of crystalline domains in the PEU. Figure 5 shows the ability to adjust the drug release rate by changing the degree of crystallinity of the PEU RCM.

There were minor changes in the TAF release rate after sterilization. We were unable to detect polymer molecular weight changes after e-beam sterilization. The implants experience a temperature increase during sterilization that may subtly affect polymer structure and drug diffusivity. If a 20% increase in release rate is unacceptable after e-beam sterilization, one could use a polymer blend or a thicker membrane to reduce the release rate and engineer the drug release change from radiation exposure into the implant design process using the design principles evident in Figs. 5 through 7. More importantly, these implants had no detectable drug substance loss from e-beam sterilization and display equivalent release profiles after periods of thermal stress.

We found that our implants did not have a strict zero-order release profile, which we would expect from ideal reservoir devices that can obtain steady states for a long duration. However, the cumulative release plots are quite linear (Fig. 8c) from day 7 to 91 (Pearson r = 0.998). Nonetheless, our data show that implant geometry driven changes in the release rate can be approximated reasonably well by using the steady-state model for a single implant composition (Eq. 1). After testing a single implant composition, the impact of making the membrane thicker or increasing the diameter of the implant can be approximated using the steady-state model. Figure 7 shows the release rate of TAF is nearly linear across the cylindrical implants tested likely because of the boundary conditions of the equation change relatively slowly throughout the duration of the device, allowing us to approximate the process using a pseudo-steady state. Furthermore, the concentration of the dissolved drug is generated by a reservoir of the insoluble drug in the implant, and changes in release rate are therefore likely to be slow relative to drug diffusion through the membrane. We found the model useful in the design of this implant system (Fig. 7) to estimate the size of an implant to achieve a particular target daily dose before we manufactured the implant. (27)

A constant activity source is assumed to use eq. 2, meaning that the drug concentration remains constant at the inner surface boundary (41). The loading density of TAF is high, ~700 mg of TAF per ml of internal volume. The drug occupies the majority of the internal volume of the implant, and the internal volume only moderately increases from swelling and hydration, by ~30% by mass (Fig. 4b). This leaves relatively little aqueous solution to solubilize TAF that saturates at 10 mg/ml of TAF. We estimate that only several hundred micrograms of TAF are solubilized in the core of the device after a week in IVRT media. These data together strongly suggest that a saturated solution of TAF develops in the implant and is maintained by a reservoir of undissolved drug substance for several months. Furthermore, Table II shows the large majority of the drug present in the implant at 60 days is the parent molecule. In total, the data support the development of pseudo-steady state concentration of TAF at the inner wall of the membrane and further support using the model to predict average release rates with a series of analogous implant formulations of varying dimensions.

TAF presents experimental and practical complexities as the molecule, like many prodrugs, is unstable in aqueous media. Other reports on TAF implants neither provide mass balance, extraction data, nor mention hydrolysis of TAF in release media. The release rate from the described implants does appear to become more variable after day 60, but until then, only half of the TAF load has been released, and the drug content is ~90% TAF in the core of the device. This variability in release early and late in the implant duration may be due to osmotically induced flows while the implant is swelling, core dissolution dynamics, variable TAF activity, and codiffusion of multiple species as the drug reservoir becomes depleted.

## Conclusion

In this paper, we describe the manufacturing and properties of a modular, tunable, long-acting implant to deliver TAF. We have demonstrated that the TAF release rate deterministically scales with implant geometry, and can be adjusted by the composition of the membrane. These heat-sealed pellet-in-a-tube designs can be easily engineered to obtain a range of TAF doses over long durations. To extend the duration of the type of device described here, more work could be done on the pellet formulation to provide a more constant prodrug activity in the core. Here pellet size, hydrophobicity, excipients, and pellet manufacturing technique could play a significant role in improving the formulation. Rohan and Shah have recognized this need and have investigated formulations of TAF that have reduced the rates of hydrolysis of TAF (42). Overall, the pellets -in-a-tube design is a flexible approach to long-acting delivery systems, and these data warrant further investigation with other drugs, formulations and polymer membranes.

### ACKNOWLEDGMENTS AND DISCLOSURES

We thank Gilead Sciences, Inc., for generously supplying the drug substance. We also thank Jim Rooney, M.D., Vahid Zia, Ph.D., and Erik Berglund, M.D., Ph.D. of Gilead Sciences Inc. for reviewing the manuscript. This work was funded by The National Institute of Allergy and Infectious Diseases of the National Institutes of Health under award number UM1 AI120184 to PFK.

## Electronic supplementary material

ESM 1 (DOCX 189 kb)

## Abbreviations

AIDS: Acquired immunodeficiency syndrome
ARV: Antiretrovirals
DDR: Drawn down ratio
DRB: Draw ratio balance
e-beam: Electron beam
f2: Similarity factor
FTC: Emtricitabine
GPC: Gel permeation chromatography
HIV: Human immunodeficiency virus
HPLC: High-performance liquid chromatography
IVRT: *In vitro* release testing
MgSt: Magnesium stearate
M_n_: Number average molecular weights
M_w_: Weight average molecular weights
NaCl: Sodium chloride
PBS: Phosphate-buffered saline
PEU: poly(tetramethylene oxide) based poly(ether) urethane
PEVA: poly(ethylene-co-vinyl acetate)
PK: Pharmacokinetic
PMPA: 9-(2-Phosphonomethoxypropyl)adenine
PrEP: Pre-exposure prophylaxis
PTMO: poly(tetramethylene oxide)
RCM: Rate-controlling membrane
RITC-Dx: Rhodamine B isothiocyanate-dextran 70 kDa
TAF: Tenofovir alafenamide hemifumarate
TDF: Tenofovir disoproxil fumarate
TFV: Tenofovir
TFV-DP: Tenofovir diphosphate
