# Supplementary material for: Design of a Drug-Eluting Subcutaneous Implant of the Antiretroviral Tenofovir Alafenamide Fumarate
Source: Pharm Res. 2020 Apr 15;37(4):83. doi: 10.1007/s11095-020-2777-2 (PMC7160069; doi:10.1007/s11095-020-2777-2)
Supplement: Supplementary file 1 — (DOCX 189 kb) [file 11095_2020_2777_MOESM1_ESM.docx]

PHAM-D-19-00437

**Supplemental Material**

**Figure S1.** Pellet mass and TAF strength consistency in a batch of pellets. The standard deviations for pellet mass and TAF strength were ± 0.32 mg and ± 0.38 mg, respectively.

**Figure S2. Kinetics of TAF hydrolysis in PBS buffer.**Initial TAF concentration was 10 µg/ml, PBS solution [PO_4_^3-^] = 11.9 mM [NaCl]= 137 mM; pH 7.4 phosphate-buffered saline adjusted to pH 7.0 with 1N HCl and pH 8.0 with 1 N NaOH. Reaction temperature 37 °C at 80 RPM on an orbital shaker. TAF concentration measured by HPLC described in the methods.

**Figure S3.** **The effect of e-beam sterilization on release rate**. Average daily release of TAF equivalent per cm lumen length from implants with Tecoflex™ EG-85A RCM (2.2 mm OD, 150 µm thickness) with core formulation 96:2:2 TAF:NaCl:MgSt before (**△,** n=5) and after (▲, n=9) e-beam sterilization. Error bars represent standard deviation.

**Figure S4.** **The dissolution of TAF implants after storage at various temperatures.** Average daily release of TAF equivalent per cm lumen length from implants with Tecoflex™ EG-85A RCM (2.2 mm OD, 150 µm thickness, 1.6 cm lumen length) with core pellet composition of 96:2:2 TAF:NaCl:MgSt at a zero time point (▲) and after storage at -20 °C for 1 year (🞼), room temperature for 1 year (🞇), and 40°C for 6 months (🞑). Error bars represent standard deviation (n=5).

**TABLES**

**Table S-I.** Extrusion conditions for tubing of various polymers and geometries

| **Polymer** | **Outer diameter (mm)** | **Wall thickness (µm)** | **Zone temperatures^a^ (hopper to die, °C)** | **Drawn down ratio** | **Draw ratio balance** |
| --- | --- | --- | --- | --- | --- |
| Tecoflex™ EG-85A | 2.2 | 150 | 140/185/185/165/140 | 26.44 | 0.99 |
| Tecoflex™ EG-85A | 2.2 | 200 | 135/160/180/180/135 | 26.44 | 0.99 |
| Tecoflex™ EG-85A | 2.2 | 360 | 135/160/180/180/135 | 7.77 | 1.03 |
| Tecoflex™ EG-85A | 3.6 | 150 | 135/165/195/190/140 | 10.80 | 1.00 |
| Tecoflex™ 75:25 EG-85A:93A | 2.2 | 150 | 135/160/180/180/135 | 27.53 | 1.00 |
| Tecoflex™ 50:50 EG-85A:93A | 2.2 | 150 | 135/160/180/180/135 | 27.53 | 1.00 |
| Tecoflex™ 25:75 EG-85A:93A | 2.2 | 150 | 145/165/190/180/130 | 19.47 | 0.99 |
| Tecoflex™ EG-93A | 2.2 | 150 | 140/165/195/165/135 | 19.40 | 0.95 |
| Celanese PEVA | 2.2 | 150 | 170/170/170/160/100 | 26.44 | 0.99 |
| 87.5:12.5 Tecoflex™ EG-85A: Tecophilic™ HP-60D-20 | 2.2 | 150 | 150/170/190/175/120 | 19.47 | 0.95 |

^a^The first three zone temperatures are for the barrel, and the last two are for the die.

**Table S-II.** Geometries and loading for all implants

| **PEU** | **Outer diameter (mm)** | **Wall thickness (µm)** | **Lumen length (cm)** | **TAF loading (mg; means ± SD)** | **Unit Length TAF loading (mg/cm)** | **TAF *in vitro* unit length release rate (µg/cm/day; means ± SD)^a^** | **Study** |
| --- | --- | --- | --- | --- | --- | --- | --- |
| Tecoflex™ EG-85A | 2.2 | 150 | 0.8 | 16.8 ± 0.4 | 21.0 | 102 ± 20 | Figure 6, 7 and 8; polymer blend; salt concentration^c^ |
| Tecoflex™ EG-85A | 2.2 | 150 | 1.6 | 33.9 ± 0.3 | 21.2 | 112 ± 24 | Figure 6, 7 and 8; stability; sterilization^c^ |
| Tecoflex™ EG-85A | 2.2 | 150 | 3.7 | 87.3 ± 0.6 | 23.6 | 105 ± 26 | Figures 6 and 7 |
| Tecoflex™ EG-85A | 2.2 | 200 | 1.0 | 16.6 ± 0.3 | 16.6 | 64 ± 16 | Figures 6 and 7 |
| Tecoflex™ EG-85A | 2.2 | 360 | 1.9 | 18.8 ± 1.6 | 9.9 | 22 ± 8 | Figures 6 and 7 |
| Tecoflex™ EG-85A | 3.6 | 150 | 3.8 | 271.3 ± 2.4 | 71.4 | 269 ± 67 | Figures 6 and 7 |
| 75:25 Tecoflex™ EG-85A:93A | 2.2 | 150 | 0.9 | 15.8 ± 0.3 | 17.5 | 67 ± 17 | Polymer blend |
| 50:50 Tecoflex™ EG-85A:93A | 2.2 | 150 | 0.9 | 16.0 ± 0.3 | 17.7 | 41 ± 14 | Polymer blend |
| 25:75 Tecoflex™ EG-85A:93A | 2.2 | 150 | 1.5 | 24.6 ± 0.5 | 16.4 | 23 ± 8 | Polymer blend |
| 0:100 Tecoflex™ EG-85A:93A | 2.2 | 150 | 1.1 | 15.8 ± 0.3 | 14.4 | 15 ± 5 | Polymer blend |
| PEVA (28% vinyl acetate content) | 2.2 | 150 | 1.3 | 24.3 ± 0.6 | 18.7 | 0.6 ± 1 | Polymer blend |
| 87.5:12.5 Tecoflex™ EG-85A:Tecophilic™ HP-60D-20 | 2.2 | 150 | 1.4 | 24.8 ± 0.5 | 17.7 | 95 ± 23 | Polymer blend |
| Tecoflex™ EG-85A | 2.2 | 150 | 1.2 | 27.9 ± 0.3 | 23.2 | 65 ± 17 | Salt concentration |
| Tecoflex™ EG-85A | 2.2 | 150 | 1.2 | 26.9 ± 0.9 | 22.4 | 99 ± 27 | Salt concentration |
| Tecoflex™ EG-85A^b^ | 2.2 | 150 | 1.2 | 25.1 ± 0.6 | 20.9 | 187 ± 57 | Salt concentration |
| Tecoflex™ EG-85A | 2.2 | 150 | 1.6 | 34.0 ± 0.3 | 21.3 | 100 ± 23 | Stability |
| Tecoflex™ EG-85A | 2.2 | 150 | 1.6 | 34.0 ± 0.4 | 21.3 | 95 ± 20 | Stability |
| Tecoflex™ EG-85A | 2.2 | 150 | 1.6 | 34.0 ± 0.1 | 21.3 | 87 ± 14 | Stability |
| Tecoflex™ EG-85A | 2.2 | 150 | 1.6 | 33.6 ± 0.2 | 21.0 | 93 ± 17 | Sterilization |

^a^Average over days 7 to 91

^b^Average over days 7 to 63 due to depletion of contents

^c^These implants are called generation A implants in Su et al. (27)

**Table S-III.** Table of individual masses of TAF and its related species measured in release media

each day for the same set of implants shown in **Figure 8B**

| **Day** | **TAF (µg/day)^a^** | **PMPA monoamidate (µg/day)** | **monophenyl PMPA (µg/day)** | **TFV (µg/day)** |
| --- | --- | --- | --- | --- |
| 1 | 18.8 ± 3.2 | 4.3 ± 0.8 | 0.0 ± 0.0 | 0.4 ± 0.2 |
| 2 | 29.7 ± 5.4 | 3.7 ± 3.9 | 0.0 ± 0.0 | 0.0 ± 0.0 |
| 3 | 34.8 ± 3.9 | 6.7 ± 2.4 | 0.0 ± 0.0 | 0.0 ± 0.0 |
| 5 | 31.7 ± 21.3 | 16.0 ± 14.1 | 0.0 ± 0.0 | 10.6 ± 22.0 |
| 7 | 65.0 ± 11.6 | 10.8 ± 7.1 | 0.0 ± 0.0 | 3.2 ± 2.8 |
| 10 | 69.1 ± 5.6 | 17.6 ± 1.2 | 0.0 ± 0.0 | 0.4 ± 0.2 |
| 14 | 99.2 ± 8.8 | 6.4 ± 3.7 | 0.0 ± 0.0 | 3.3 ± 1.2 |
| 21 | 91.1 ± 4.5 | 19.3 ± 3.1 | 0.0 ± 0.0 | 2.2 ± 1.1 |
| 25 | 96.8 ± 3.1 | 16.2 ± 0.7 | 0.0 ± 0.0 | 6.2 ± 0.8 |
| 30 | 102.7 ± 4.9 | 10.2 ± 0.0 | 0.0 ± 0.0 | 9.6 ± 0.7 |
| 37 | 82.9 ± 4.6 | 21.3 ± 3.4 | 0.0 ± 0.0 | 2.6 ± 0.1 |
| 42 | 106.6 ± 7.2 | 21.7 ± 1.4 | 0.0 ± 0.0 | 7.8 ± 0.6 |
| 49 | 81.0 ± 7.0 | 20.5 ± 1.1 | 0.0 ± 0.0 | 0.0 ± 0.0 |
| 56 | 110.8 ± 6.1 | 15.4 ± 1.7 | 0.0 ± 0.0 | 0.0 ± 0.0 |
| 63 | 67.1 ± 4.6 | 35.6 ± 2.9 | 0.0 ± 0.0 | 3.5 ± 0.5 |
| 70 | 99.2 ± 25.9 | 59.9 ± 19.3 | 0.0 ± 0.0 | 0.0 ± 0.0 |
| 77 | 107.6 ± 6.7 | 3.5 ± 0.3 | 0.0 ± 0.0 | 3.2 ± 2.2 |
| 85 | 82.9 ± 8.5 | 62.1 ± 9.4 | 0.0 ± 0.0 | 4.0 ± 0.2 |
| 91 | 99.4 ± 4.9 | 43.3 ± 11.0 | 0.0 ± 0.0 | 4.9 ± 2.1 |
| 98 | 138.3 ± 8.2 | 32.8 ± 4.0 | 0.0 ± 0.0 | 5.6 ± 0.4 |
| 105 | 118.8 ± 17.6 | 27.5 ± 2.1 | 0.0 ± 0.0 | 7.4 ± 1.0 |
| 112 | 81.4 ± 40.4 | 28.7 ± 12.7 | 1.4 ± 0.0 | 7.4 ± 0.5 |
| 119 | 9.3 ± 17.1 | 9.3 ± 13.3 | 0.0 ± 0.0 | 1.2 ± 1.3 |
| 126 | 0.0 ± 0.0 | 1.6 ± 0.0 | 0.0 ± 0.0 | 12.2 ± 8.5 |
| 133 | 2.4 ± 1.5 | 0.6 ± 1.7 | 1.3 ± 0.0 | 4.3 ± 0.3 |
| 140 | 0.0 ± 0.0 | 0.7 ± 0.0 | 0.0 ± 0.0 | 8.0 ± 2.0 |
| 147 | 0.0 ± 0.0 | 3.6 ± 0.0 | 0.0 ± 0.0 | 4.9 ± 2.2 |
| 154 | 0.0 ± 0.0 | 0.0 ± 0.0 | 0.0 ± 0.0 | 1.7 ± 0.9 |
| 164 | 0.7 ± 0.2 | 7.9 ± 3.0 | 0.0 ± 0.0 | 0.0 ± 0.0 |
| 169 | 0.0 ± 0.0 | 0.0 ± 0.0 | 0.0 ± 0.0 | 0.7 ± 0.9 |
| 175 | 0.0 ± 0.0 | 1.4 ± 0.5 | 0.0 ± 0.0 | 0.7 ± 0.8 |

^a^Using tenofovir alafenamide hemifumarate molecular weight
